# Under the Earth

**Published:** 1891-09

**Authors:** 


### UNDER THE EARTH.

The workmen in the deepest mines of Europe swelter in almost intolerable heat, and yet they never penetrate over one 7-1000th part of the distance from the surface to the centre of the earth.

In the lower levels of some of the Comstock mines the men fought scalding water, and could labor only three or four hours at a time until the Sutro tunnel pierced the mines and drew some of the terrible heat, which had stood at 120.

The deepest boring ever made, that at Sperenberg, near Berlin, penetrates only 4,172 feet, about 1,000 feet deeper than the famous artesian well at St. Louis.

While borings and mines reveal to us only a few secrets relating solely to the temperature and constitution of the earth for a few thousand feet below the surface, we are able, by means of volcanoes, to form some notion of what is going on at a greater depth.

There have been many theories about the causes of volcanoes, but it is now generally held that, though they are produced by the intense heat of the interior of the earth, they are not directly connected with the molten mass that lies many miles below the immediate sources of volcanic energy.

Everybody knows that many rocks are formed on the floor of the ocean, and it is found that a twentieth to a seventieth of their weight is made up of imprisoned water. Now, those rocks are buried in time under overlaying strata, which serve as a blanket to keep the enormous heat of the interior.

This heat turns the water into superheated steam, which melts the hardest rock, and when the stream finds a fissure in the strata above it it breaks through to the surface with terrific energy, and we have a volcano.

We find that these outpourings that have lain for countless ages many thousands of feet below the surface are well adapted to serve the purposes of man. Many a vineyard flourishes on the volcanic ashes from Vesuvius, and volcanic mud has clothed the hills of New Zealand with fine forests, and its plains with luxuriant verdure.

The most wonderful display of the results of volcanic energy is seen in the northwestern corner of our own land, a region of lofty forests and of great fertility.—*Toronto Truth.*

